# Mental Health Policies in Low and Lower Middle-Income Countries (LLMICs): A Narrative Synthesis

**DOI:** 10.1192/bjo.2024.355

**Published:** 2024-08-01

**Authors:** Usman Arshad, Imran Chaudhry, Nasim Chaudhry, Rachel Jenkin, Nusrat Husain

**Affiliations:** 1 Pakistan Institute of Living and Learning, Karachi, Pakistan; 2 University of Manchester, Manchester, United Kingdom; 3 Kings College London, London, United Kingdom; 4 Mersey Care NHS Trust Foundation, Prescot, United Kingdom

## Abstract

**Aims:**

Background: Mental health policy is crucial for enhancing mental health and well-being. Despite the significant contribution of mental disorders to the global burden of disease, 68% of the countries possess a comprehensive mental health policy. This review aimed to identify similarities and differences between low-income countries' (LICs) and lower middle-income countries' (LMICs) mental health policies, along with key gaps, limitations, and strengths, to inform Pakistan's mental health policy.

**Methods:**

We conducted searches on Google, the WHO Mental Health Atlas, and the country's Ministry of Health website for mental health and general health policies. Recent mental health policies were included from LMICs that were available in English, whether published or unpublished. Scholarly articles, commentaries, books, and health policies that did not address mental health were excluded. Data extraction covered document title, policy status, country, policy formulation process, human resources, suicide prevention, finances, health service delivery, governance, leadership, involvement of ministries, and implementation plans. We synthesized the data through a comparative narrative review in both text and tables.

**Results:**

Fifty percent (8/16) of LICs and sixty-five percent (17/26) of LMICs have health and mental health policies in English. These policies cover topics like psychiatric disorders, psychotropic drugs, forensic mental health, substance abuse disorders, and communicable and non-communicable diseases. Approximately 65% of LMICs' policies outline the structure of their federal or national government, and 59% provide information on provincial and local government structures. Most LICs include their vision, mission, and objectives in their policies.

**Conclusion:**

Mental health is often neglected in the healthcare policies of LICs and LMICs. To reduce the burden of mental illness and prevent self-harm, suicide, and substance misuse disorders, the implementation of evidence-based mental health policies in line with the Sustainable Development Goals (SDGs) is crucial.

